# High-responsivity InSe/TaSe_2_ photodetectors integrated on low-loss silicon nitride waveguides

**DOI:** 10.1039/d5na00119f

**Published:** 2025-09-29

**Authors:** Maaz Ahmed Qureshi, Fooqia Khalid, Md Gius Uddin, Abde Mayeen Shafi, Isaac Doughan, Janvit Tippinit, Faisal Ahmed, Xiaoqi Cui, Matthieu Roussey, Harri Lipsanen, Zhipei Sun, Markku Kuittinen

**Affiliations:** a Center for Photonics Sciences, University of Eastern Finland 80100 Joensuu Finland maaz.qureshi@uef.fi markku.kuittinen@uef.fi; b Department of Electronics and Nanoengineering, Aalto University Espoo Finland uddinm2@aalto.fi zhipei.sun@aalto.fi

## Abstract

Two-dimensional materials integrated with waveguides present a promising platform for the development of high-performance on-chip photodetectors due to their exceptional optoelectronic properties. In this study, we demonstrate both simulated and experimental results by fabricating and characterizing an InSe/TaSe_2_ heterojunction photodetector on a low-loss silicon nitride waveguide. The fabricated device achieves a high responsivity of 2.54 AW^−1^, an external quantum efficiency (EQE) of 592%, a noise equivalent power (NEP) of 12 nW, and a noise power detection ratio (NPDR) of 25.8 PAW^−3^ at a source-drain voltage (*V*_sd_) of 2 V. In particular, the responsivity of the InSe/TaSe_2_ heterojunction exceeds that of the reference InSe-based photodetector by more than 50%. Additionally, when illuminated with laterally incident light through the waveguide, the photodetector exhibited significantly higher responsivity compared to the normal-incidence configuration, achieving approximately a 20-fold enhancement as a result of stronger light-matter interactions and the intrinsic properties of the heterostructure. These findings highlight the potential of on-chip heterojunction-based photodetectors for applications in sensing, imaging, and integrated communication systems.

## Abstract 2

Waveguide-integrated InSe/TaSe_2_ heterostructure photodetectors exhibit high responsivity and low noise, enabled by enhanced light-matter interaction.

## Graphical abstract

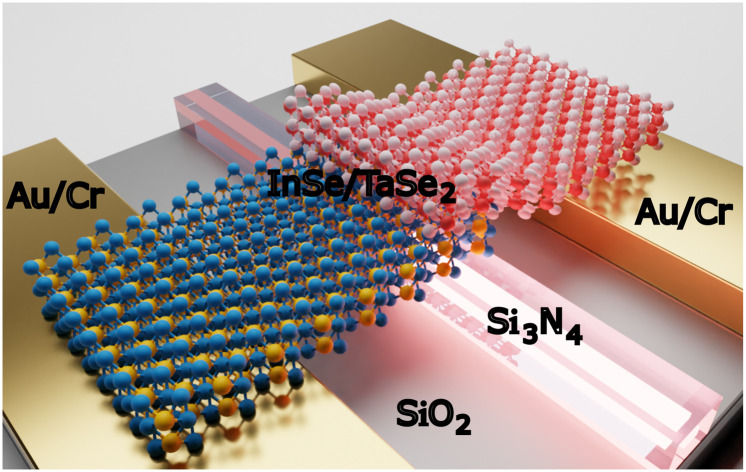

## 1 Introduction

During the last decade, integrated photonics and two-dimensional (2D) materials^1,2^ have gained immense importance for promising optoelectronics and photonics applications^3^ in the fields of data processing,^4^ quantum computing,^5–7^ and optical communication,^8–10^ to name a few. Integrating 2D materials on a silicon photonics chip can lead to high-performance devices such as photodetectors, optical modulators, and light sources that can work on a wide range of wavelengths.^11^ Photodetectors based on graphene,^12–14^ the most widely studied 2D material, have generally weak photosensitivity, and optical responsivity of such devices is usually limited to only a few mAW^−1^.^15^ To combat this challenge, semiconducting 2D materials such as MoS_2_,^16–18^ MoTe_2_,^19,20^ MoSe_2_,^21^ and others are explored with a diverse range of energy bandgaps from 0.3 to >2.2 eV to obtain strong light-matter interaction and responsivity to a few tens of mAW^−1^ over a wide range of electromagnetic spectrum.

Although the semiconducting 2D materials showed an increase in responsivity, it was proposed to integrate these 2D materials with integrated photonics structures such as waveguides, photonic crystals, plasmonic structures, and microcavities to further enhance the light absorption in the 2D materials.^22–25^ Among these photonic structures, silicon nitride (Si_3_N_4_) waveguides have proven to be a highly desirable platform due to their excellent compatibility with CMOS processes, wide transparency window (400–5000 nm), and ultra-low propagation loss. These properties make Si_3_N_4_ an ideal material for low-loss, scalable integration with 2D materials in sensing and optoelectronic applications.^26–29^ Such integration of 2D materials with waveguides significantly increases the light-matter interaction and photoresponsivity of the material to a few hundred of mAW^−1^.^25,30^ Although remarkable photoresponsivities of over 1000 AW^−1^ are also achieved in some devices, it can be overlooked that these responsivities generally benefit from doping and photogating effects.^16,31,32^ Some of the earlier reported articles of 2D-waveguide photodetectors demonstrated with graphene at the wavelength of 1550 nm,^33^ MoS_2_ at 532 nm,^34,35^ MoTe_2_ at 1300 nm (ref. 1) and at 1550 nm,^36^ black phosphorous at 1575 nm (ref. 37) and at 2000 nm,^38^ InSe at 976 nm (ref. 11) of wavelength. Here, we focused our research on layered indium selenide (InSe) due to its promising applications in photodetectors, optical sensors, and other photonics components.^11,39,40^

InSe has recently gained interest due to its electrical, optical, and mechanical properties.^41^ Because of the high carrier mobilities of the material (over 10^3^ cm^2^ V^−1^ s^−1^ at room temperature and 10^4^ cm^2^ V^−1^ s^−1^ at cryogenic temperatures),^41^ InSe-based photodetectors can demonstrate very high responsivity over broad wavelength ranges from the visible to the near-infrared spectrum.^42^ In contrast to many traditional transition metal dichalcogenides (TMDCs), InSe exhibits a tunable bandgap crossover from indirect to direct upon increasing its thickness and has an out-of-plane absorption dipole (OP).^43^ In addition, excitons are strongly transferred from the valence band to the conduction band when the polarization of the incident light is perpendicular to the plane of the InSe material.^43–45^ One photonic component that can provide such properties of incident light is an optical waveguide where the polarized component of the field can be set normal to the plane of the waveguide, and an InSe flake placed on top of it. Moreover, integrating InSe material with a layered TMDC 2H-TaSe_2_ (tantalum diselenide) can provide effective charge carrier transport in the InSe/TaSe_2_ junction, which can ideally further enhance the light-matter interaction in the heterostructure with its strong photoluminescence and light absorption properties caused by its interband transitions.^46^ Thus, strong light-coupling and absorption can be expected if we integrate multilayer InSe and TaSe_2_ flakes with an optical waveguide.

In this article, we demonstrate an ultrasensitive InSe/TaSe_2_ van der Waals heterojunction photodetector on top of Si_3_N_4_ waveguides. The device operates under low bias and exhibits a responsivity reaching over 2.54 AW^−1^ at visible wavelength that is over 6 times higher compared to the other previously reported InSe-based devices (∼0.38 AW^−1^ (ref. 11) and ∼0.11 AW^−1^ (ref. 47)), and over 5000 times enhanced compared to the first graphene photodetector^13^ (∼0.5 mAW^−1^). The improved responsivity of our device is a consequence of better carrier mobility, low-contact resistance, low-loss Si_3_N_4_ waveguide, strong light-matter coupling, and reduced contamination in the device. The fabricated device with the integration of 2D material and Si_3_N_4_ waveguide offers new avenues for on-chip photodetectors.

## 2 Photodetector design and experimental results

InSe/TaSe_2_ heterostructure and InSe crystals integrated with Si_3_N_4_ waveguides are simulated to demonstrate the light absorption in these flakes using ridge waveguide structures. Mode shift and light absorption in the flakes are compared to comprehend the effect of introducing InSe and TaSe_2_ in the device. The heterostructure photodetector consists of a multilayer InSe that is first transferred onto a waveguide and the metal contacts. Then, a TaSe_2_ flake is transferred and stranded on top of an InSe flake and the metal contacts on the other side of the waveguide.

### 2.1 Design and simulations

**Fig. 1:**
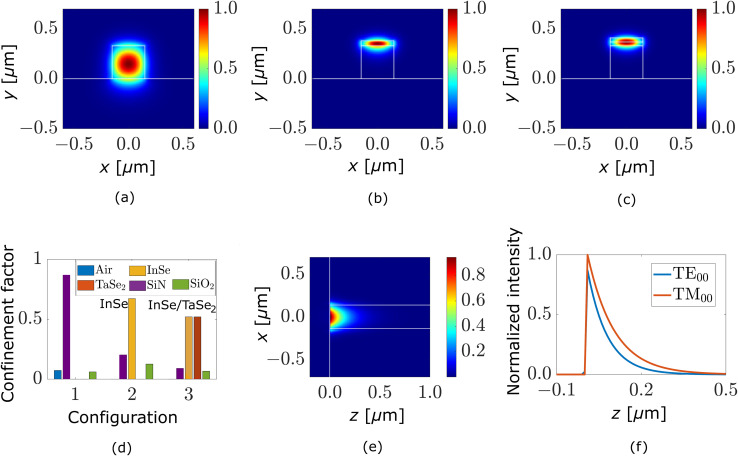
Electric field |E| mode-profile simulation of InSe/TaSe_2_ heterostructure on a Si_3_N_4_ waveguide. A cross-section of a single-mode profile is shown in (a) with no 2D materials, (b) with a 20 nm InSe flake (c) with a 20 nm InSe flake and 20 nm TaSe_2_ flake on the top of the waveguide. (d) Confinement factor of a mode in waveguide, 2D materials, surrounding regions for (a) represented by configuration 1, (b) represented by configuration 2, and (c) represented by configuration 3. This graph shows that over 70% of light is absorbed in InSe in configuration 2, while over 90% of light is absorbed in InSe/TaSe_2_ heterostructure in configuration 3. (e) Top view of the InSe/TaSe_2_ heterostructure configuration represented in (c). (f) Illustrates the effective absorption of light in InSe/TaSe_2_ flakes within a short length of the waveguide for this heterostructure configuration for TE and TM fundamental modes.

A single-mode Si_3_N_4_ waveguide (Si_3_N_4_ core: 0.33 μm thick and 0.30 μm wide) is simulated to observe the propagation of the fundamental transverse electric (TE) mode in a waveguide. Fig. 1a shows the cross-section of a single mode confined in the core of a Si_3_N_4_ waveguide when the propagation of light is in the *z*-direction (into the plane of the page). Fig. 1b and c represent the mode profile with only InSe on top of the waveguide and both InSe and TaSe_2_ on top of the waveguide, respectively. The optical mode shifts upwards into the flakes at the top of the waveguides, indicating that the light-matter interaction increases and more light is absorbed in the flakes.

Fig. 1d summarizes the confinement factors which describe that the mode in Fig. 1a is 90% confined in the Si_3_N_4_ waveguide, while in Fig. 1b the mode is 70% confined in the InSe and only 30% confined in the Si_3_N_4_ waveguide and surroundings (cladding and substrate region). And in Fig. 1c, the total confinement of the InSe and TaSe_2_ increases to 90% (55% in InSe and 35% in TaSe_2_) and only 10% to the surroundings. Through the simulation results, it can be expected that more absorption should occur in the InSe/TaSe_2_ heterostructure compared to other configurations. Fig. 1e shows a simulation of the top view of the waveguide for InSe/TaSe_2_ heterostructure, and it can be observed that light is rapidly absorbed into the top layers of flakes. Fig. 1f shows a simulation of the effective absorption of light in the InSe/TaSe_2_ with the propagation distance for TE and TM fundamental modes. It can be observed that both the TE and TM modes are fully absorbed in a short length. Therefore, for the selection and transfer of our flakes experimentally, the lengths of the flakes can be in the range of a few microns to achieve maximum absorption of light in the flakes to develop a small footprint photodetector.

### 2.2 Experimental setup

**Fig. 2:**
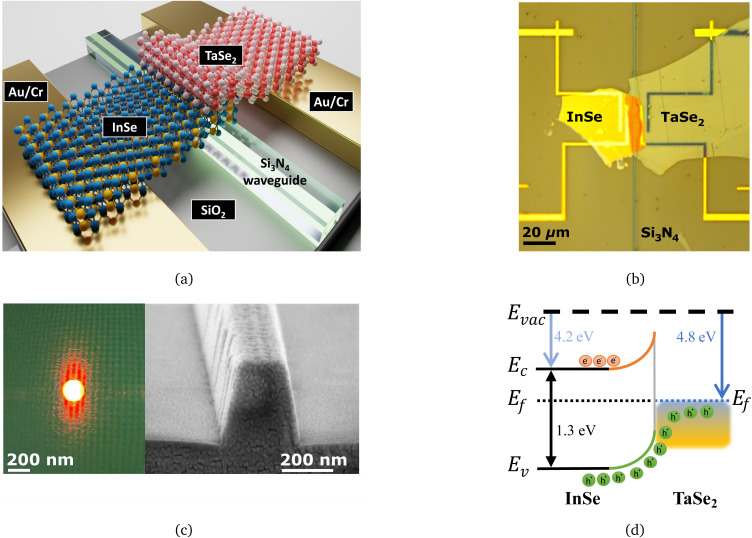
Characterization of InSe/TaSe_2_ heterostructure on a Si_3_N_4_ waveguide. (a) 3D schematic diagram of InSe/TaSe_2_ heterostructure transferred on top of a Si_3_N_4_ waveguide and pre-patterned Au/Cr metal contacts. (b) Optical microscope image of the InSe and TaSe_2_ flakes transferred on the fabricated Si_3_N_4_ waveguide and metal contacts. A multilayer InSe flake is transferred on the waveguide and on the electrodes on the left side of the waveguide, while a multilayer TaSe_2_ flake is transferred on top of the already transferred InSe (on the waveguide) and on the electrodes on the right side of the waveguide. The overlapping part (in orange color) represents the heterojunction between the two materials. (c) Optical mode and SEM image of a cross-section for a single-mode waveguide. The dimensions of a waveguide cross-section are approximately 0.3 μm (width) × 0.33 μm (height). (d) Band diagram of the multilayer InSe/TaSe_2_ heterojunction at equilibrium giving rise to the photocurrent.

Si_3_N_4_ waveguides and metal contacts are pre-patterned on a SiO_2_/Si substrate by dry etching and physical vapor deposition. InSe and TaSe_2_ flakes are transferred one after another on the waveguide and metal contacts at the last step of device fabrication to reduce contamination, the time to prevent material deterioration, and improve the electrical and optical response of the device. Details of the fabrication process and materials are explained in Section 4. The waveguides are characterized using a custom-built setup introduced in Fig. S1 in the SI. A 3D schematic of the device concept is represented in Fig. 2a, showing the junction of InSe/TaSe_2_ on a waveguide. As light is coupled laterally into the waveguide, it is subsequently absorbed in the InSe and TaSe_2_ crystals through the evanescent tails of the waveguide mode that overlap with the crystals. The length of the InSe/TaSe_2_ device (represented in orange color in Fig. 2b) is approximately 40 μm on a 0.30 μm wide waveguide. The simulations suggested that a few micrometers of the length of the crystals should be enough to absorb all of the light in the waveguide, allowing us to maximize the generated photocurrent while keeping the device size to a minimum.

It is important to note that due to the refractive index mismatch, a certain amount of optical reflection can occur at the interface of a bare waveguide and the photodetector junction region. However, this reflection effect is significantly reduced by an evanescent field coupling (rather than normally directed light) between the Si_3_N_4_ waveguide and the thin InSe/TaSe_2_ flake, as shown in Fig. 2a and b, allowing efficient light absorption at the coupling interface. In addition, the flakes uniformly cover the waveguide surface, as shown in Fig. 2b, which reduces the abrupt changes in optical impedance and thus reduces Fresnel reflections at the interface.^48^ Such minimal back-reflection losses under such coupling interfaces have also been studied in similar waveguide-integrated 2D material photodetectors.^37,49,50^

Single mode obtained at the output of the waveguide without any material on top of the waveguide, and an SEM image of the cross-section of a waveguide is shown in Fig. 2c. The fabricated waveguide core is approximately 0.33 μm thick and 0.30 μm wide. The side walls of the waveguide are slanted due to the plasma etching process. As the fibered laser output diameter is approximately 2 μm, a 3 μm wide taper is used at the waveguide input to inject light from a fibered laser by a butt-coupling method. Hence, a wider taper is beneficial in properly injecting the light into the waveguide with less insertion loss. We obtained propagation and insertion losses of 1.05 dB cm^−1^ and 12.09 dB. Details of waveguide loss measurements are shown in SI Fig. S1. The propagation losses are slightly increased by fabrication-induced imperfections, such as sidewall roughness from reactive ion etching, as clearly seen in the SEM image in Fig. 2c. These surface and sidewall irregularities increase the scattering losses in high-confinement Si_3_N_4_ waveguides.^28,51^ In this study, the photodetectors were designed and fabricated to prioritize effective 2D material integration and device functionality, rather than minimizing waveguide losses alone, to demonstrate the feasibility of hybrid photodetector performance. Further optimization of the material transfer and etching processes is expected to reduce both propagation and insertion losses.

Fig. 2d illustrates the band diagram of multilayer InSe with TaSe_2_ at equilibrium. The integration of semi-metallic TaSe_2_ results in a conduction band offset relative to InSe. This offset creates an energy barrier that blocks electron transfer from InSe to TaSe_2_, while the offset of the valence band facilitates the transfer of the hole from InSe to TaSe_2_ across the metal–semiconductor interface, allowing efficient hole extraction.^52,53^ This behavior is depicted in Fig. 2d, where electron flow is restricted and hole transport is favored due to the alignment of the band. Furthermore, this offset, combined with TaSe_2_'s semi-metallic nature, can significantly reduce contact resistance at the interface, improving the overall efficiency of charge injection and extraction.^53^ The formation of a 2D InSe/TaSe_2_ Schottky barrier modulates charge carrier dynamics through thermionic emission and tunneling processes, leading to improved photodetection due to efficient carrier separation and reduced recombination.^53^ These theoretical insights are validated by our electrical and optical characterizations presented in the subsequent sections.

### 2.3 Photodetectors measurements

The analysis of photodetector measurements is divided into three categories: InSe-based configuration, InSe/TaSe_2_ heterojunction configuration, and finally, a comparison of these configurations. The measurements include the results with the laterally incident and normally incident light.

#### 2.3.1 InSe-based photodetector configuration

A photodetector was fabricated as described in Section 2.2. The Raman spectrum and the photoluminescence response of InSe flake, as well as the flake thickness measurement by atomic force microscopy (AFM), are shown in the SI, Fig. S2 and S3, respectively (in the same figures, the Raman spectrum and the thickness measurement result for the TaSe_2_ flake are also shown). The Raman spectra show high crystallinity of the samples, and the AFM measurements show that the InSe and TaSe_2_ flakes are about 20 nm thick, consisting of 25–30 atomic layers of the material. The photoluminescence spectrum of InSe has a center wavelength of 991 nm when an excitation wavelength (*λ*) of 532 nm is used.

**Fig. 3:**
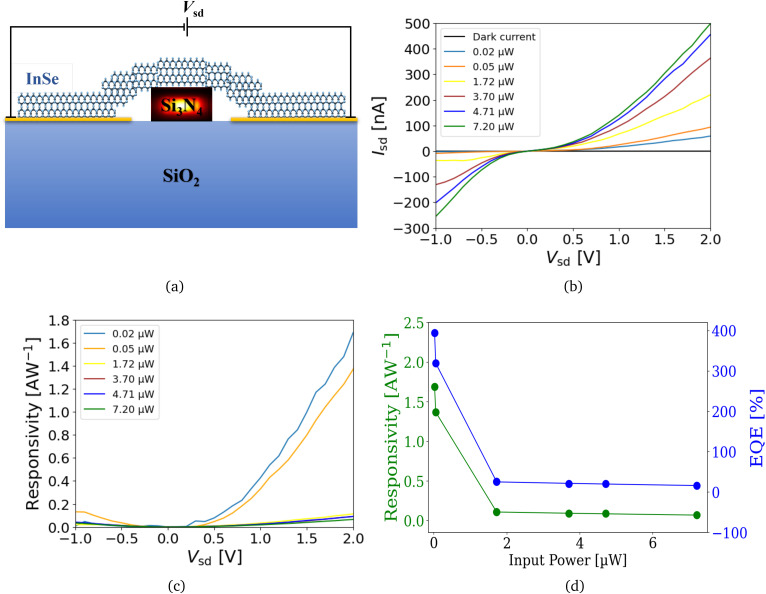
Electrical characteristics and photoresponse of InSe device with laterally incident light in the waveguide. All measurements are conducted at a *V*_g_ of 0 V. (a) 2D schematic illustration of the device with InSe and waveguide cross-section along with the electrical configuration. (b) *IV*-characteristics and photocurrent generated for varying optical powers. (c) Responsivity dependence on *V*_sd_ in the device. (d) Responsivity and EQE of the device for different optical powers of laser light coupling laterally in the waveguide at *V*_sd_ of 2 V.

The exfoliated InSe flake was transferred onto a waveguide, and a schematic cross-section of the device is shown in Fig. 3a. For this configuration, the *IV* characteristic, *i.e.*, the current *I*_sd_*versus* the source-drain voltage *V*_sd_, was measured when light was injected laterally through the waveguide. The measurement results are shown in Fig. 3b. The corresponding results for normal incident light are shown in SI, Fig. S4. A *V*_sd_ of −1 to 2 V is applied at a gate voltage (*V*_g_) of 0 V using source measurement units (SMUs) with different input powers of green light (532 nm laser). Although the devices are tested at *V*_g_ from −20 V to +120 V and *V*_sd_ from −5 V to +5 V, we have only reported the results for *V*_g_ at 0 V and *V*_sd_ from −1 V to +2 V. The low power measurements ensure the intrinsic state of the device without external influences and maintain the stability of the device in applications where low power consumption is essential.

Fig. 3b shows that by increasing the optical power of the incident light from 0.02 μW to 7.20 μW and changing *V*_sd_ from −1 V to 2 V, the *I*_sd_ in the device increases. The dark electrical response, *i.e.*, the response without incident light, for the InSe-based device is shown on a more accurate scale in SI, Fig. S5a. Since some laser light is inevitably lost due to the insertion and propagation losses in a waveguide before the light reaches the device, we estimated the actual power received by the photodetector by applying the transmission loss equation of our fabricated waveguide, as detailed in SI Fig. S1c.

The increase in the electrical response is a result of the generation of photocurrent (*I*_ph_) in the InSe device with the input light. Photocurrents at *V*_g_ of 0 V and *V*_sd_ from −1 V to 2 V in the InSe device are shown in SI, Fig. S6(a and c). We observed the highest photocurrent to be of over 400 nA at *V*_sd_ of 2 V and *V*_g_ of 0 V for the light of 7.20 μW optical power (*P*_opt_) received by the photodetector in lateral light coupling. The responsivity (*R*) of our device is then calculated for the variation of *V*_sd_ as shown in Fig. 3c. The highest value of 1.69 AW^−1^ is obtained at *V*_sd_ of 2 V. Fig. 3d illustrates the variation of responsivity and external quantum efficiency (EQE) with waveguide optical power. Both parameters, calculated using eqn (1), exhibit a decreasing trend as optical power increases, likely due to the optical saturation effect in the device.1
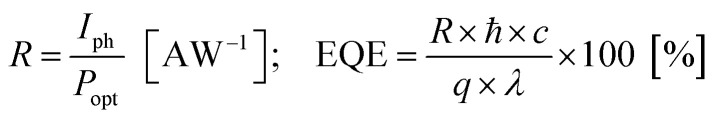
where ℏ is Planck's constant, *c* is the speed of light, *q* is the elementary charge, and *λ* is the incident wavelength.

The optical saturation effect occurs due to the limited number of available charge carriers, which limits further photocurrent generation at higher excitation levels.^41,50^ In InSe photodetectors, this effect occurs when the power of the incident light exceeds the ability of the flake to efficiently extract and transport charge carriers, mainly due to its relatively low carrier mobility, defect-related recombination, and ultrathin structure.^41,52,54^ Consequently, the photocurrent response begins to saturate even at moderate optical intensities. Strong excitonic interactions further influence this behavior, and such optical saturation has also been widely observed in other 2D material-based photodetectors.^50,55^ The corresponding results of responsivity for normal incident light are shown in the SI, Fig. S4(c and d).

#### 2.3.2 InSe/TaSe_2_ heterojunction photodetector configuration

**Fig. 4:**
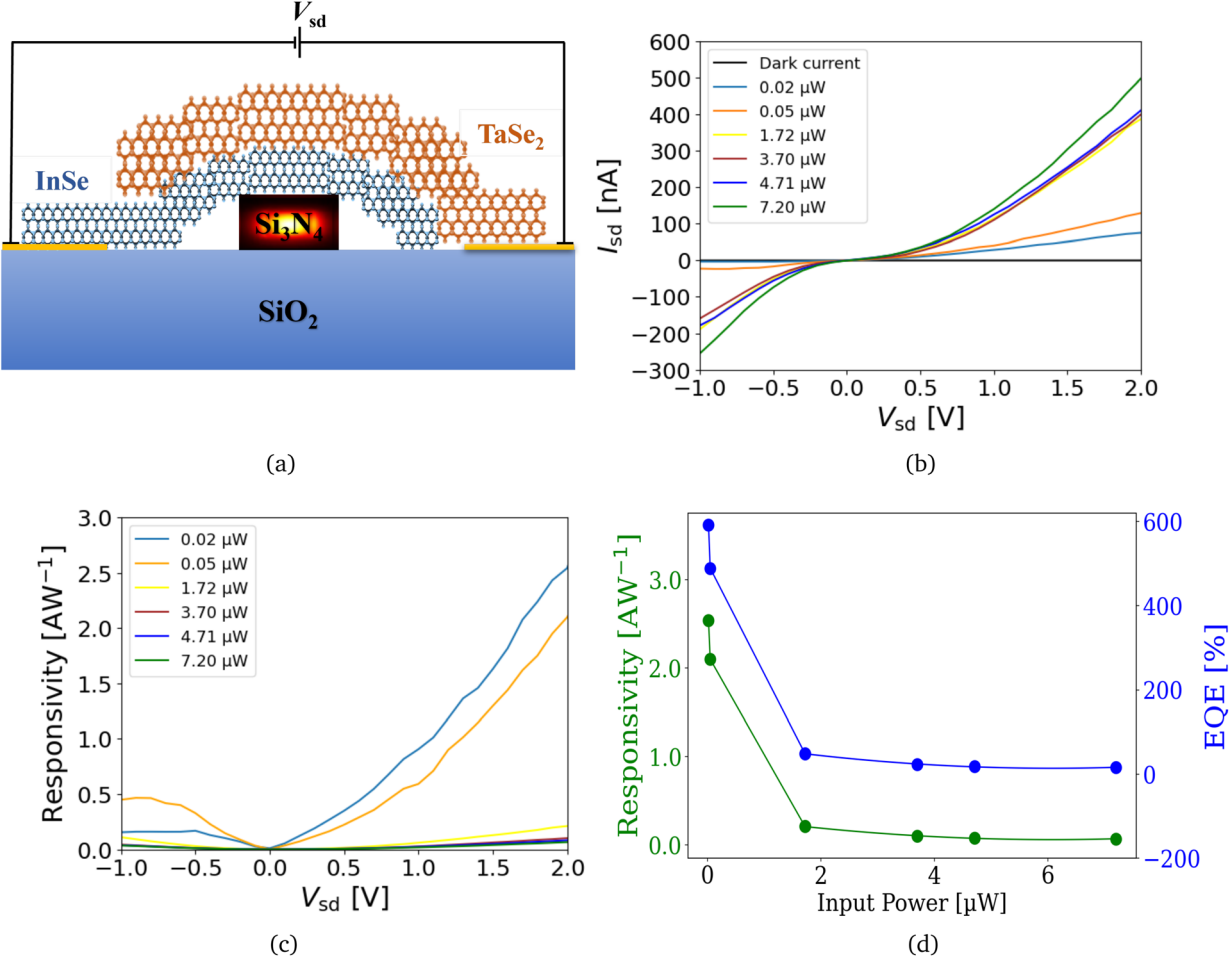
Electrical characteristics and photoresponse of InSe/TaSe_2_ device with laterally incident light in the waveguide. All measurements are conducted at a *V*_g_ of 0 V. (a) 2D schematic illustration of the device with InSe/TaSe_2_ and waveguide cross-section along with the electrical configuration. (b) *IV* characteristics and photocurrent generated for varying optical powers. (c) Responsivity dependence on *V*_sd_ in the device. (d) Responsivity and EQE of the device of 2 V for different optical powers of laser light coupling laterally in the waveguide at *V*_sd_ of 2 V.

A device similar to the one in Section 2.3.1 is utilized to measure the performance when an exfoliated flake of TaSe_2_ is transferred on an already placed flake of InSe on a waveguide, as shown in Fig. 4a. Since the flake of the InSe/TaSe_2_ device is the same as in our previously discussed InSe-based device, all the factors contributing to the electrical response of the two devices (*i.e.*, flake thickness and length, waveguide dimensions and transmission losses, and flake-metal contact resistances) are kept the same.

A similar trend in the electrical response and responsivity of the InSe/TaSe_2_ heterostructure device is observed, which is comparable to the InSe-based device as shown in Fig. 4b. An increase in the *I*_sd_ is observed with the increase in *V*_sd_ and optical powers. Photocurrents in the device are shown in SI, Fig. S6(b and d). Experiments are conducted with the same set of optical power values, so we can compare the results precisely. Again, in Fig. 4c and d, similar trends are observed for responsivity with the variation of *V*_sd_ and optical powers. However, the actual highest responsivity obtained with InSe/TaSe_2_ heterostructure device is 2.54 AW^−1^ and an EQE of 592% is obtained at a *V*_sd_ of 2 V. Since we kept all or most of the factors contributing to the responsivities of the two devices the same, we hereby report experimentally that the addition of TaSe_2_ crystal to fabricate a InSe/TaSe_2_ heterostructure has resulted in an increase of responsivity in our InSe/TaSe_2_-based photodetector by over 50%.

For normal incident light, the corresponding electrical responses of the InSe/TaSe_2_ heterostructure device are shown in SI Fig. S7, and responsivity results are shown in SI, Fig. S7(c and d). SI Fig. S7e shows the photocurrent mapping in the device as light is coupled vertically from the top. It can be observed that maximum current is generated in the InSe/TaSe_2_ heterostructure, as indicated by the red region. While the dark electrical response for the InSe/TaSe_2_ device is shown on a more accurate scale in SI Fig. S5b, photocurrents in the device are shown in SI Fig. S6d.

Compared to previously reported photodetectors based on similar materials,^11,47^ this InSe/TaSe_2_ heterostructure device showed one of the highest responsivity values achieved. The exceptional performance can be attributed to several factors: the fabrication of low-loss Si_3_N_4_ waveguides, the efficient transfer of InSe and TaSe_2_ flakes, and the incorporation of TaSe_2_, which significantly enhanced charge carrier transport in the InSe/TaSe_2_ device, leading to improved responsivity. The electrical response and responsivity trends for laterally incident light in the waveguide are presented in Fig. 3 and 4 for both InSe-based and InSe/TaSe_2_ heterostructure devices. These trends are consistent with those observed for normal incident light, as shown in SI, Fig. S4 and S7, although the responsivity values for laterally incident light are over an order of magnitude higher. The underlying reasons for this enhanced responsivity are discussed in Section 2.4.

#### 2.3.3 Configuration comparison

**Fig. 5:**
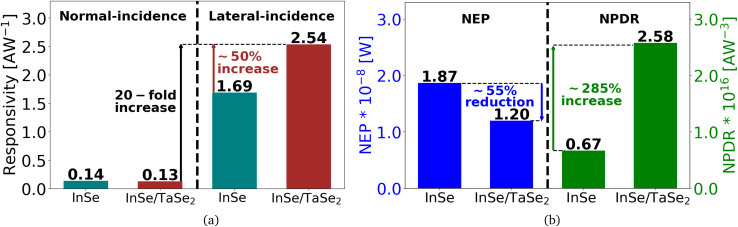
Comparison of responsivity, NEP, and NPDR for different device configurations with normally incident light coupling and laterally incident light coupling through the waveguide at *V*_g_ of 0 V and *V*_sd_ of 2 V. (a) Increase in the responsivity of 20-fold in InSe/TaSe_2_ heterostructure and 10-fold in InSe-based device is observed for light coupling laterally through the waveguide compared to the normally incident light coupling. (b) Reduction of over 55% for NEP and a 285% increase for NPDR for InSe/TaSe_2_ heterojunction compared to only InSe-based photodetector for laterally incident light. The increase in responsivity is not only due to the increase in the light-matter interaction of the materials with evanescent tails of the guided mode but also to the enhancement of photoluminescence, as the electric field component in the laterally injected light in the waveguide aligns with the intrinsic out-of-plane dipole moment in InSe. In addition, a 50% increase in responsivity is observed in the InSe/TaSe_2_ heterostructure compared to the InSe-based crystal in the waveguide configuration due to the effective charge carrier transport in the InSe/TaSe_2_ heterojunction.

Fig. 5 presents a bar chart comparing the responsivity, Noise Equivalent Power (NEP), and Noise Power Detection Ratio (NPDR) for different light propagation configurations. The measurements were conducted under consistent conditions, with *V*_g_ set to 0 V and *V*_sd_ set to 2 V across all structural device configurations. The first two bars in Fig. 5a depict the responsivity of the InSe and InSe/TaSe_2_ devices under normal incidence illumination, while the last two bars show the responsivity when light is coupled laterally through the waveguide. In the waveguide configuration, the light interacts with the 2D material through the evanescent tails of the guided mode. This results in a significantly longer interaction length compared to the normal incidence configuration, thereby enhancing the absorption within the flake.

Moreover, InSe crystal has an out-of-plane dipole moment aligned with an electric field in the laterally injected light that further enhances the photoluminescence and absorption of light in the crystal which results in an increase of photocurrent generation and responsivity of the device. The graph vividly illustrates that the devices measured with the waveguide configuration have significantly higher responsivity than the devices with the normally incident light. Specifically, it is estimated that there is a 10-fold increase in the responsivity of the InSe-based device and a 20-fold increase in the responsivity of the InSe/TaSe_2_ heterostructure device with the laterally incident light coupling compared to the normally incident light coupling. This 20-fold increase in the responsivity response underlines the significance of using optical waveguides as a light-guiding medium for photodetectors based on 2D materials.

Furthermore, the graph also suggests an increase of 50% in the responsivity with an InSe/TaSe_2_ heterostructure device compared to the pure InSe-based device in the waveguide configuration due to the effective transport of charge carriers in the InSe/TaSe_2_ heterojunction. This result is in accordance with the simulation results discussed in Section 2.1, where it is suggested that the InSe/TaSe_2_-based device can have significantly more light confinement and absorption compared with the InSe-based device. To the best of our knowledge, such a high responsivity (2.54 AW^−1^) and high EQE (592%) of InSe/TaSe_2_ based photodetectors or similar devices have not yet been reported.

Fig. 5b illustrates a significant improvement in device performance, with a 55% reduction in NEP and a 285% increase in NPDR when transitioning from an InSe-based photodetector to an InSe/TaSe_2_-based structure. Consistent with the responsivity measurements, the NEP and NPDR values, calculated using eqn (2), were recorded at *V*_g_ of 0 V and *V*_sd_ of 2 V.2
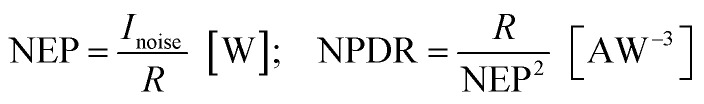


Fig. S8 shows the NEP and NPDR response of our photodetectors with the voltage sweep from −1 V to +2 V. The results highlight the superior performance of the InSe/TaSe_2_-based photodetectors compared to the InSe-based device throughout the voltage range. A benchmark comparison is presented in SI Fig. S9a (tabulated summary) and Fig. S9b (visual scatter plot), showing that our InSe/TaSe_2_ heterojunction device achieves a peak responsivity of 2.54 AW^−1^, surpassing most previously reported devices and standing out among the best-performing photodetectors. These results underscore the competitive performance and practical potential of our fabricated device platform for next-generation, high-responsivity photonic sensing based on 2D material integration.

### 2.4 Justification of high responsivity in InSe/TaSe_2_

Compared with normally incident light coupling, we demonstrated an order-of-magnitude increase in the responsivity and effective light absorption for laterally incident light in the waveguide. Several mechanisms account for this, and they are summarized below.

**Fig. 6:**
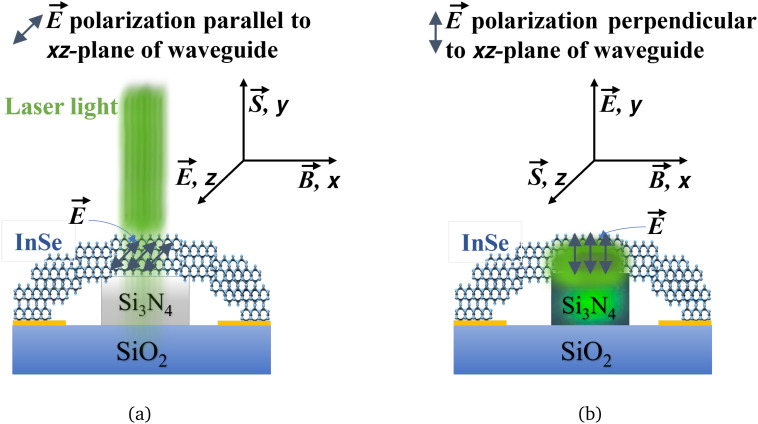
Effect of dipole orientation in InSe crystal on Si_3_N_4_ waveguide when excited light polarization 
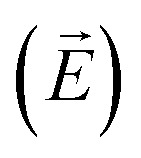
 is (a) parallel (normally incident light coupling) (b) perpendicular (laterally incident light coupling) to the *xz*-plane of the waveguide. Blue arrows indicate the dipole orientation of atoms in InSe in both configurations. 
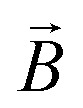
 and 
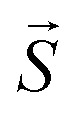
 are the magnetic field and propagation direction of the field, respectively. Green light in the InSe crystal over the Si_3_N_4_ waveguide in (b) indicates the shifted optical mode from the waveguide onto the top InSe crystal that interacts with the out-of-plane dipoles to enhance light-matter interaction in the InSe crystal.

When dealing with a stack of 2D material sheets like InSe, the main effect comes from the dipole orientation, which is directly related to the polarization of the electric field 
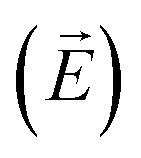
 concerning the geometry of the material film. Fig. 6 illustrates this effect for a film deposited on top of a waveguide. In the case of a normally incident light coupling as in Fig. 6a, there is no polarization effect since both s and p polarizations are equivalent. Namely, 
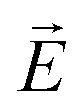
 is within the plane of the film, which is leading to the weakest absorption.^56^ However, when light is injected in the waveguide as in Fig. 6b, *i.e.*, laterally incident light coupling, 
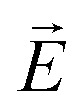
 can be either parallel to the film (quasi-TE modes) or perpendicular to the film (quasi-TM modes).^45^ In the second case, the absorption is prominent and leads to a drastic enhancement when 
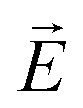
 aligns with the out-of-plane dipoles of InSe.^41,45,57^

The second aspect to consider is simply the length of the interaction of light with the absorbing medium. In the case of normally incident light coupling, the amount of light absorbed is proportional to the surface area of the incident beam multiplied by the thickness of the absorbing medium. In the case of laterally incident light coupling in a waveguide, the absorption is proportional to the thickness of the film multiplied by the width of the waveguide, multiplied by the length of the absorbing layer covering the waveguide. The simulations presented in Fig. 1f show clearly that after just a few micrometers, all light is absorbed for both polarizations, with, however, a clear difference between the TE and TM modes. It is important to note that only a part of the mode, that is, related to the confinement factor of 
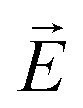
 in the absorbing region, is absorbed.

Furthermore, the InSe/TaSe_2_ heterojunction demonstrates superior performance due to efficient photon absorption in InSe due to its direct band gap, high absorption coefficient, and Schottky contact along the InSe/TaSe_2_ interface, as shown in Fig. 2d.^45,58^ Semi-metallic TaSe_2_ forms a Schottky-type contact with InSe, allowing efficient hole extraction due to favorable offset of the valence band, while offset of the conduction band restricts electron transfer, further reducing recombination losses.^52,59,60^ This carrier separation, together with the built-in electric field, improves photocurrent generation and responsivity, consistent with observations in similar 2D heterojunction devices.^52,61–63^ Moreover, on-chip integration with a low-loss silicon nitride waveguide improves the light-matter interaction, further improving the responsivity and performance of the device.^64^

Recently, it has been shown that Raman and photoluminescence are enhanced by almost 10-fold due to the dipole orientation engineering of InSe on nanowires.^43^ The angle between the nanowire axis and normally incident light is varied, and the maximum response is obtained as the angle decreases to zero, that is, when the incident light is parallel to the nanowire axis. We used a similar conceptual idea, but instead of implementing nonluminous nanowires and changing the angle of light coupling from normally to laterally, we fabricated optical waveguides to directly guide the light into the waveguide and the InSe crystal laterally. Since in our waveguide configuration, 
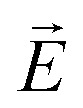
 can be perpendicular to the film (that is, 
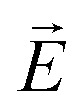
 aligns with the out-of-plane dipoles of the InSe crystal), we can expect efficient light absorption that should enhance the photoresponse in the InSe. The results in Fig. 5 show the difference between the configurations and demonstrate an obvious enhancement of the responsivity due to light absorption, dipole orientation, and efficient carrier separation.

The main aim of this work was to demonstrate a highly sensitive photodetector with enhanced responsivity and external quantum efficiency enabled by waveguide-integrated 2D materials. While the results of our InSe/TaSe_2_ photodetector highlight improved noise and sensitivity performance, a key limitation is the lack of time-resolved response measurements. Due to limitations of our system, we cannot perform the transient characteristics of photodetectors, such as the speed, rise, and fall times. However, such measurements can be a focus of future studies.

## 3 Conclusions

In conclusion, we have successfully demonstrated, for the first time, a high responsivity InSe/TaSe_2_ heterojunction photodetector on a low-loss Si_3_N_4_ waveguide. The fabrication process of the device is simple, taking advantage of the dipole orientation of InSe for effective light coupling in the material. The reported device has shown an order of magnitude enhancement in responsivity in the laterally incident light coupling (waveguide configuration) compared to the normally incident light coupling due to the out-of-plane dipole moment in the InSe crystal in parallel to the electric field component of light. The presented waveguide-coupled InSe/TaSe_2_ heterojunction achieved a high responsivity of 2.54 AW^−1^ and realized an over 50% enhancement in responsivity compared to our referenced InSe-based photodetector. Our photodetector's compact footprint and low operating voltage offer great potential for portable optoelectronic devices, particularly in areas requiring high sensitivity and high responsivity.

## 4 Device design, fabrication, materials and characterization

### 4.1 Simulation software and parameters

The simulations were performed using Ansys Optics 2023 R1.2 (MODE module) with a source wavelength of 532 nm. For Fig. 1a and c, the Finite Difference Eigenmode (FDE) solver was employed with Perfectly Matched Layer (PML) boundary conditions applied on all sides. The mesh settings consisted of a general override mesh size of 10 nm, with a finer 1 nm mesh applied within the waveguide core and a 1 μm buffer region surrounding it. Fig. 1e was simulated using the variational Finite-Difference Time-Domain (FDTD) solver to address the memory limitations of conventional FDTD methods, employing identical boundary conditions and mesh settings as in the FDE simulations. The results were then exported, analyzed and plotted using MATLAB for Fig. 1d and f.

### 4.2 Waveguide fabrication

A wafer of 525 μm thick p-type silicon (Si) with 〈100〉 orientation, 3 μm wet thermal oxide layer on Si, and 330 nm of Si_3_N_4_ layer on top by stoichiometric LPCVD is used for device fabrication. Electron-beam lithography (EBL) alignment markers are initially patterned using a positive photoresist (AR-672) followed by the development process, metal deposition, and lift-off process. A negative nLOF photoresist is then spin-coated (2000 rpm for 60 seconds to obtain a resist thickness of 500 nm), baked (110 °C for 60 seconds), and exposed by the EBL writer to pattern waveguides followed by the development process and dry etching using reactive ion etching (RIE) with CHF_3_ and O_2_ gas mixtures. The sample is further cleaned with O_2_ plasma. The fabricated waveguide cross section is 0.3 μm wide and 0.33 μm thick for a single-mode waveguide. The sample is again spin-coated and EBL patterned with the positive photoresist, followed by the development process, metal deposition (2 nm Cr/50 nm Au) using physical vapor deposition, and a lift-off process to deposit metal contacts for electrical measurements. The sample is cleaved from the side of the waveguides so that light can be injected from the fibered laser into the waveguide input using a butt-coupling method. The length of a typical waveguide in the sample is approximately 1 cm after cleaving the sides of the sample.

### 4.3 Material exfoliation and transfer

The step-by-step transfer process is explained in the literature by Kinoshita *et al.*^65^ In our study, material transfer was performed using the 2D Heterostructure Transfer System (HQ Graphene). Bulk crystals of InSe and TaSe_2_ were sourced from 2D semiconductors and exfoliated using Scotch tape by gently pressing the crystals onto the tape to form a flake-covered stamp. The stamp was further exfoliated several times to thin the layers and expand the area, then pressed onto a PDMS (polydimethylsiloxane) surface, leaving various flakes behind.

A polypropylene carbonate (PPC) solution in anisole was spin-coated onto a glass slide and soft-baked to form a thin PPC film. The PDMS with exfoliated flakes was placed onto this PPC film and removed carefully, transferring the flakes to the PPC layer. Direct contact between the tape and the PPC was avoided to prevent tearing. The flakes were inspected using an optical microscope based on color contrast, and the PPC film with the selected flake was cut and mounted on PDMS for transfer to the prepatterned Si_3_N_4_ waveguide and electrodes.

The PDMS–PPC flake stack was brought into contact with the substrate and heated to 60 °C to promote adhesion. After InSe transfer, the PPC film was dissolved in acetone, followed by an IPA rinse and nitrogen drying. The same process was repeated for TaSe_2_. After both transfers, the sample was annealed at 120 °C for 180 seconds to improve flake adhesion and remove surface contaminants.

### 4.4 Electrical and optical characterization

The sample is soldered to a printed circuit board (PCB), and Cr/Au metal contacts on the sample are connected to the metal pad of the PCB by wire bonding. The sample is fixed on a stage of a waveguide setup, and a laser light is injected from a 2 μm fiber lens into the tapered waveguide of 3 μm width input *via* a butt-coupling technique. SMUs (Keithley 2400 and 2401) are used to apply and measure the *IV* characteristics (*I*_sd_ with the variation of *V*_sd_) of the devices with and without the injected light. The power of injected light is increased to obtain the dependence of *IV* characteristics on the variation of the light power. To estimate the actual laser light power reaching the photodetector device, we measured the output power of the fiber-coupled laser and applied the transmission loss equation of our standard waveguide, as shown in SI Fig. S1. Using this method, we measured the dark and illuminated photocurrent as the laser power is varied in the waveguide.

For normally incident light coupling, we placed the sample vertically under the microscope objective (20× 0.4 NA). A frequency-doubled green Nd:YAG solid-state laser at 532 nm with a spot size of 1.6 μm was focused perpendicularly onto the sample. The same objective was used to collect the backscattered signals with a 1800 groove per mm grating. Similar to the measurements in the waveguide setup, the dark and light photocurrents were measured by varying the input laser light, and the SMUs performed a voltage sweep *V*_sd_ while measuring the *I*_sd_. The measured electrical response with the laser powers has been used to calculate the responsivity of our devices. All the measurements were conducted at room temperature, and a customized LabVIEW program was used for data acquisition.

## Author contributions

M. Q. and M. U. conceived the project under the guidance of M. K. and Z. S. M. Q. and F. K. fabricated the samples and conducted the optoelectronic measurements. I. D. performed the simulations of optical mode propagation in waveguides. M. Q. performed the data analysis and drafted the manuscript in collaboration with F. K., M. U., A. S., I. D., J. T., F. A., X. C., M. R., H. L., Z. S., and M. K. The manuscript has been reviewed by all the authors.

## Conflicts of interest

There are no conflicts to declare.

## Supplementary Material

NA-OLF-D5NA00119F-s001

## Data Availability

Data for this article is available at Zenodo (Publisher) at https://doi.org/10.5281/zenodo.14410751. Supplementary information: waveguide characterization; Raman/PL and AFM of 2D flakes; device schematics with additional dark-current/photocurrent measurements; NEP/NPDR analyses; and benchmarking *versus* prior 2D waveguide photodetectors. See DOI: https://doi.org/10.1039/d5na00119f.
