# Supplementary material for: High-responsivity InSe/TaSe2 photodetectors integrated on low-loss silicon nitride waveguides
Source: Nanoscale Adv. 2025 Sep 29;7(22):7352–62. doi: 10.1039/d5na00119f (PMC12506784; doi:10.1039/d5na00119f)
Supplement: NA-007-D5NA00119F-s001 [file NA-007-D5NA00119F-s001.pdf]

Supplementary Information (SI) for Nanoscale Advances.
This journal is © The Royal Society of Chemistry 2025

Supplementary information

High-responsivity InSe/$TaSe_2$ photodetectors integrated on low-loss silicon nitride waveguides

Maaz Ahmed Qureshi,*[a] Fooqia Khalid [b] Md Gius Uddin [b] Abde Mayeen Shafi [b] Isaac Doughan [a] Janvit Tippinit [a] Faisal Ahmed [b] Xiaoqi Cui [b] Matthieu Roussey [a] Harri Lipsanen [b] Zhipei Sun [b] and Markku Kuittinen [a]

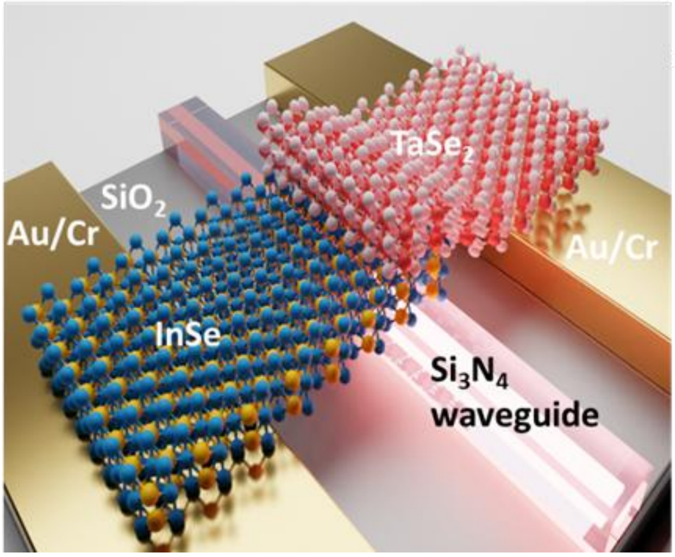

[a] Center for Photonics, University of Eastern Finland, Joensuu, Finland.
Email: *maaz.qureshi@uef.fi* , *markku.kuittinen@uef.fi*

[b] Department of Electronics and Nanoengineering, Aalto University, Espoo FI-02150, Finland.
Email: *uddinm2@aalto.fi* , *zhipei.sun@aalto.fi*

Table of content

Figure:

S1: Waveguide characterization setup and transmission loss measurements of silicon-nitride waveguides.

S2: Raman and photoluminescence of InSe and $TaSe_2$ flakes.

S3: AFM of InSe and $TaSe_2$ flakes transferred on the waveguide.

S4: Drawing and electrical response of an InSe-based device with normally incident light onto the InSe.

S5: Dark current electrical response of a pure InSe-based device and an InSe/$TaSe_2$ heterojunction device.

S6: Photocurrent generated of a pure InSe-based device and an InSe/$TaSe_2$ heterojunction device.

S7: Drawing and electrical response of an InSe/$TaSe_2$ heterostructure with normally incident light onto the heterostructure.

S8: NEP and NPDR response InSe-based and InSe/$TaSe_2$ heterojunction devices with normally incident light onto the photodetector.

S9: Comparison of our waveguide-integrated InSe-based and InSe/$TaSe_2$ heterojunction photodetector with previous photodetectors made with similar 2D materials.

S1: Waveguide characterization setup and transmission loss measurements of silicon-nitride waveguides.

(a)

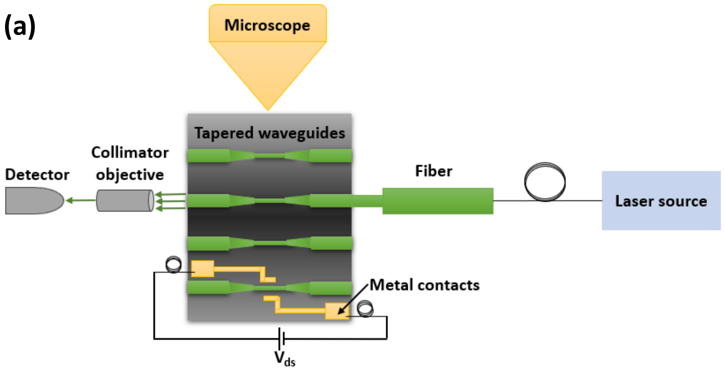

(b)

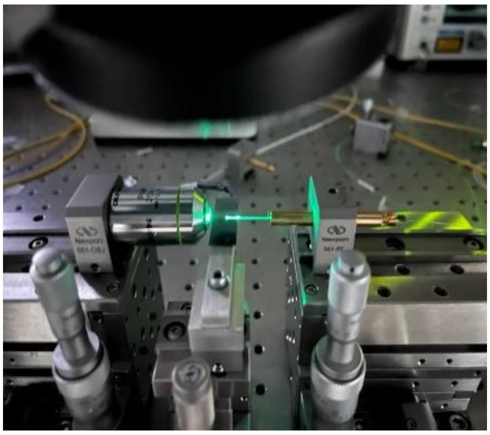

(c)

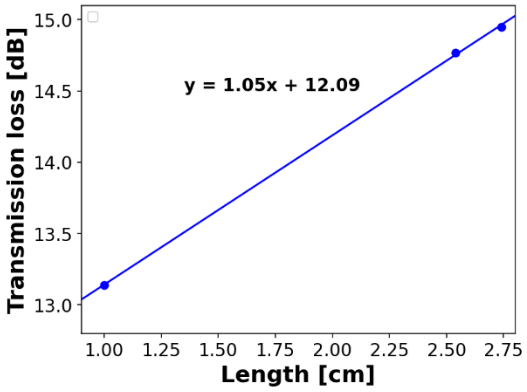

(d)

Measured Power at Laser Output (μW)	Estimated Power at Photodetector (μW)
0.41	0.02
1.03	0.05
35.49	1.72
75.92	3.70
96.65	4.71
147.74	7.20

Fig. S1: Top-view illustration and image of custom-built waveguide characterization setup with the transmission loss measurements.

A 2 µm optical fiber and 3 µm tapered waveguides are shown in green in **(a)**. A 532 nm laser source is used to inject light into the fiber and the tapered waveguide. At the output, the light is collimated by an objective and then received by the power meter or output camera to observe the optical mode. A top-view microscope is utilized to align the fiber and waveguide input until we can observe maximum light propagation in the waveguide. Metal contacts are used to conduct the electrical measurements in a 2D material with the propagation of light in the waveguide. The picture in **(b)** shows the actual experimental setup shown in (a), which shows the injected light in the waveguide. The transmission loss measurement graph is shown in **(c)**, which shows the relationship between the output power detected by the detector and the change in waveguide length. Loss measurements are made by using the cut-back method by varying the length of the waveguides. The straight line is extrapolated, and the insertion loss is found to be ~12.09 dB, while the propagation loss is ~1.05 dB/cm. Moderate insertion loss and low propagation loss ensure that most of the light would reach the photodetector device, which can support higher light-matter interaction. **(d)** Measured laser output powers and the corresponding estimated powers reaching the photodetector, calculated using the transmission loss equation (12.09 dB insertion loss and 1.05 dB/cm propagation loss). In this study, the power effectively reaching the photodetector—rather than the raw laser output—was utilized to ensure a more accurate evaluation of the device's photodetection performance.

S2: Raman and photoluminescence of InSe and $TaSe_2$ flakes.

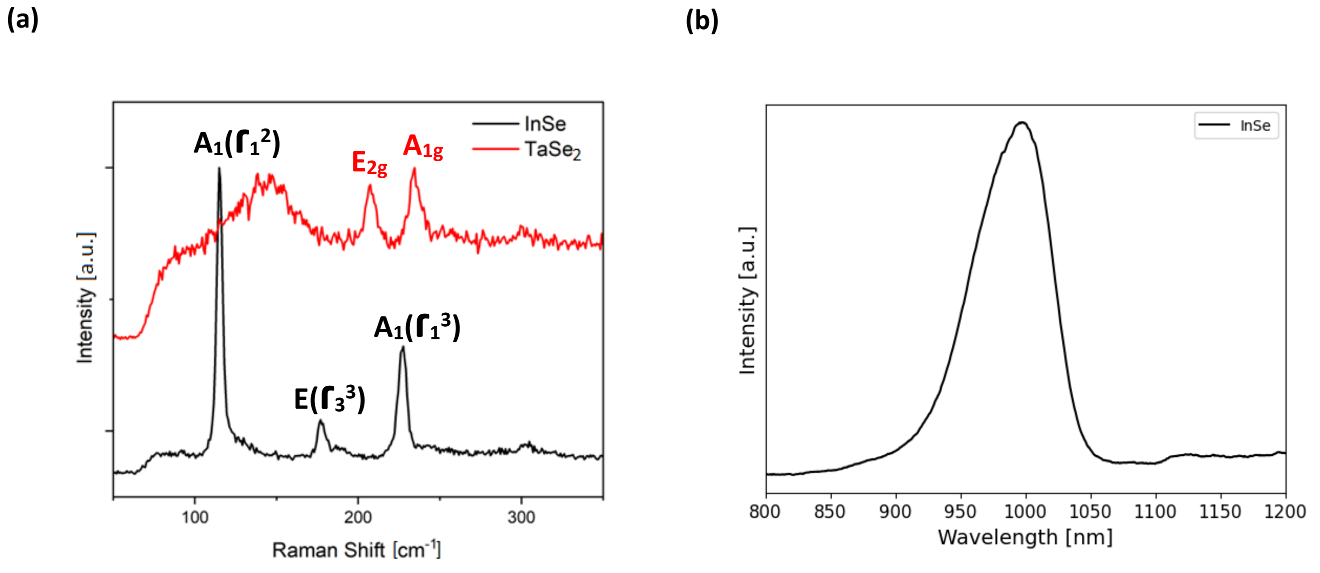

Fig. S2: Raman response of InSe and $TaSe_2$ flakes in (a) and photoluminescence response of InSe flakes in (b). The distinct phonon peaks of InSe are $A_1(\Gamma_1^2)$ at 114 cm^{-1}, $E(\Gamma_3^3)$ at 176 cm^{-1} and $A_1(\Gamma_1^3)$ at 225 cm^{-1}. The peaks of $TaSe_2$ are E_{2g} at 211 cm^{-1} and A_{1g} at 233 cm^{-1}. Peak photoluminescence intensity for InSe flake is obtained at a wavelength of 991 nm.

S3: AFM of InSe and $TaSe_2$ flakes transferred on the waveguide.

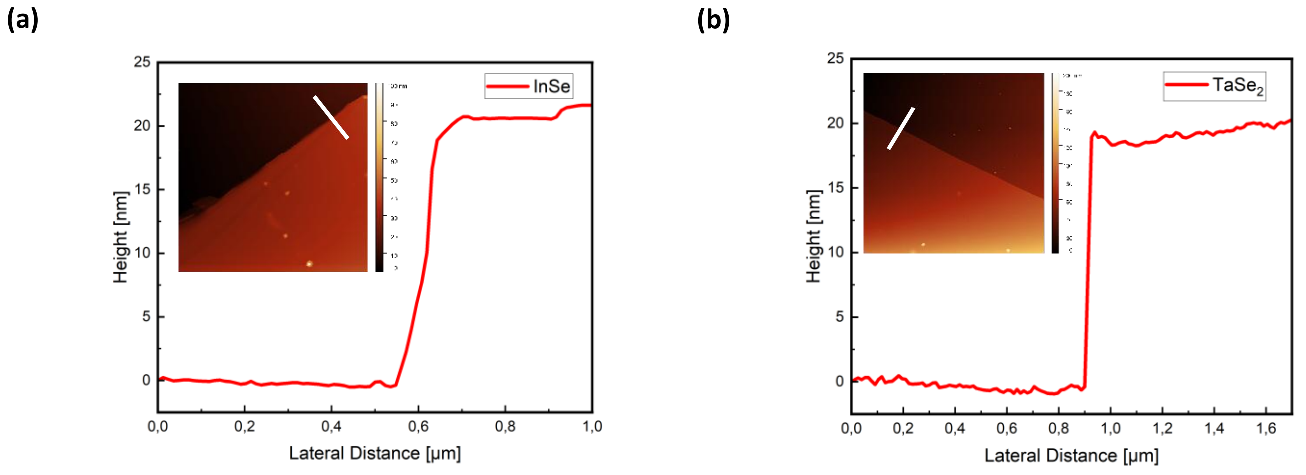

Fig. S3: AFM of InSe flake in (a) and $TaSe_2$ flake in (b). The lateral distance of the graph corresponds to the white line in the inset. The thickness of the InSe flake is estimated to be around 20 nm, which means that the InSe flake used for experimental measurements has 27 layers of thickness. The thickness of the $TaSe_2$ flake is estimated to be around 20 nm.

S4: Drawing and electrical response of an InSe-based device with normally incident light onto the InSe.

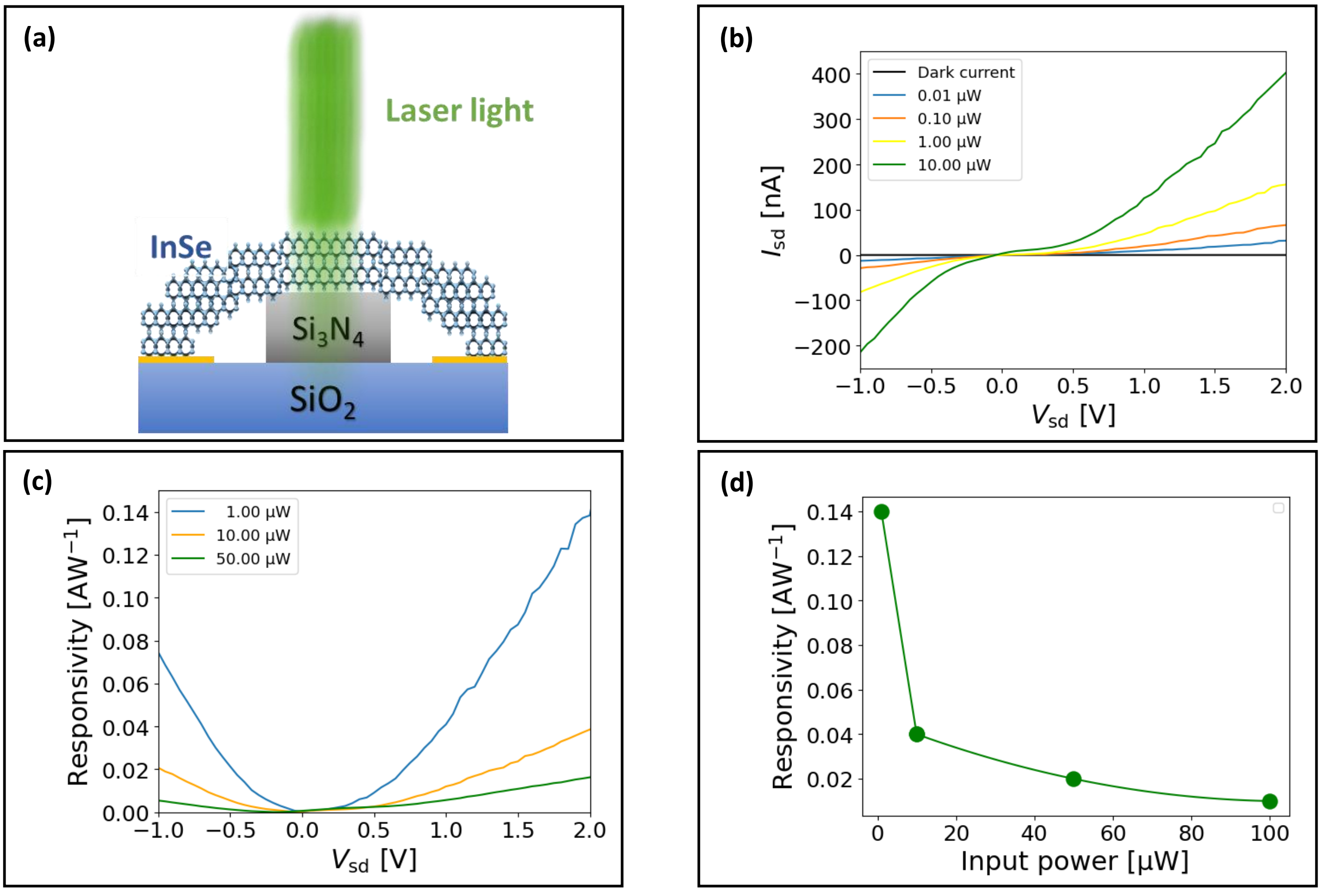

Fig. S4: Illustration and electrical response of InSe-based device with normally incident light onto the InSe. All measurements are conducted at a gate voltage of 0 V. (a) 2D schematic diagram of light incident normally from the top to the InSe device on a silicon nitride waveguide and pre-patterned Au/Cr metal contacts. (b) *IV*-characteristics and photocurrent generated for different optical powers. (c) Responsivity as a function of source-drain voltage in the device. (d) Responsivity in the device for different optical powers of green laser light incident normally from the top at a gate voltage of 0 V and source-drain voltage of 2 V.

S5: Dark current electrical response of a pure InSe-based device and an InSe/$TaSe_2$ heterojunction device.

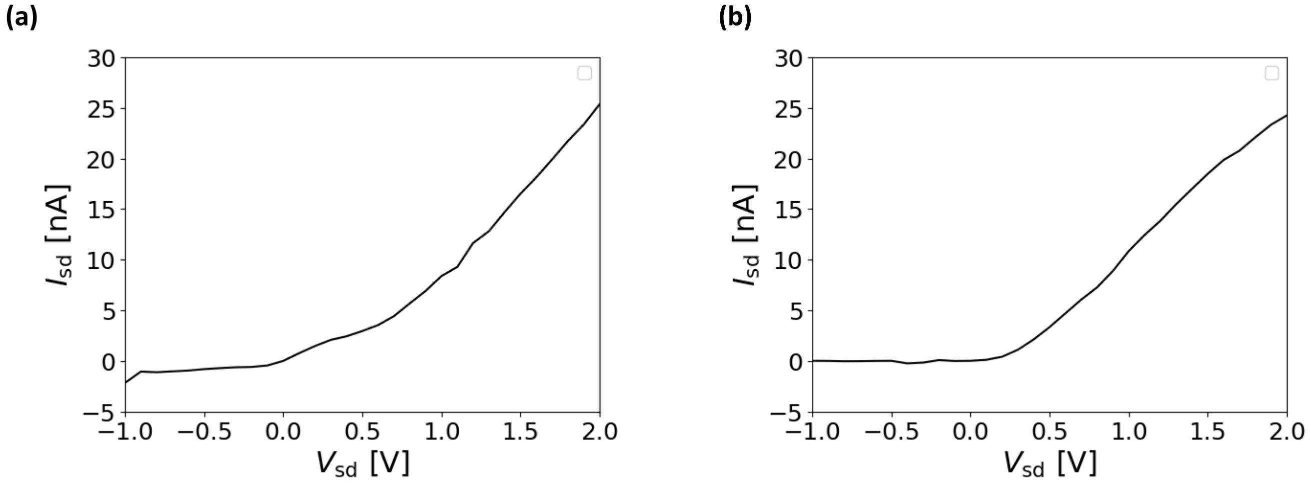

Fig. S5: Electrical response of InSe-based device and InSe/$TaSe_2$ heterojunction device. The *IV* characteristic curve of the InSe-based device is shown in (a) and of the InSe/$TaSe_2$ heterojunction in (b). These measurements are carried out under dark illumination. The *n*-type InSe shows a response at positive bias voltage which agrees with the previously reported results. The voltage is also increased beyond 5 V and at different gate voltages ranging from -20 V to 120 V and the devices remain stable even at high voltages.

S6: Photocurrent generated of a pure InSe-based device and an $InSe/TaSe_2$ heterojunction device.

(a) **InSe-based device in a laterally incident light coupling in a waveguide**

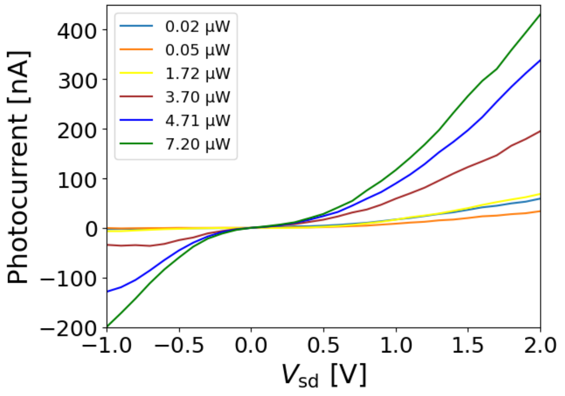

(b) **$InSe/TaSe_2$ heterojunction in a laterally incident light coupling in a waveguide**

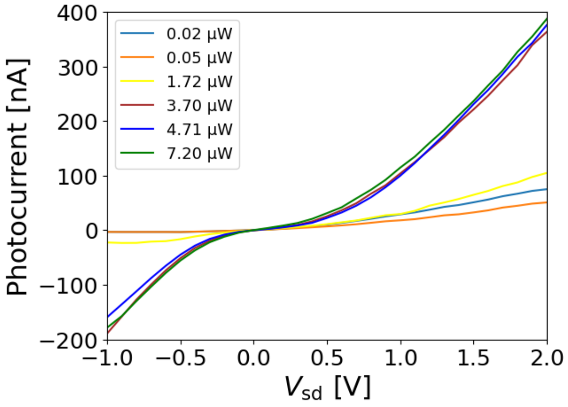

(c) **InSe-based device in a normally incident light coupling**

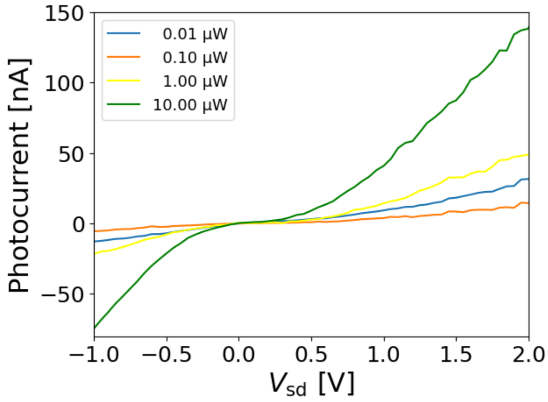

(d) **$InSe/TaSe_2$ heterojunction in a normally incident light coupling**

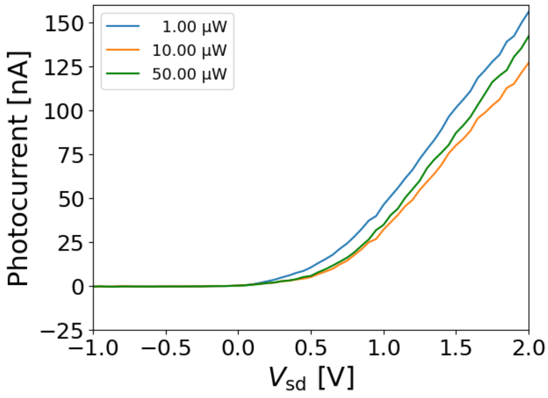

Fig. S6: Photocurrent generated in InSe-based devices and $InSe/TaSe_2$ heterojunction devices by varying the source-drain voltage for different light powers for lateral and normal light coupling.

Generation of photocurrent for InSe-based devices in (a) and (c) and $InSe/TaSe_2$ heterojunction devices in (b) and (d) for lateral and normal light coupling. The light propagates through the waveguide and the electric field is perpendicular in (a) and (b) and parallel in (c) and (d) to the direction of light and the plane of the waveguide. The perpendicular electric field interacts with the out-of-plane dipole moment of the InSe material which enhances the light absorption in the material. This is evident in the above graphs by the higher amount of photocurrent generated in lateral light coupling in waveguide configurations compared to the normal light coupling.

S7: Drawing and electrical response of an InSe/$TaSe_2$ heterostructure with normally incident light onto the heterostructure.

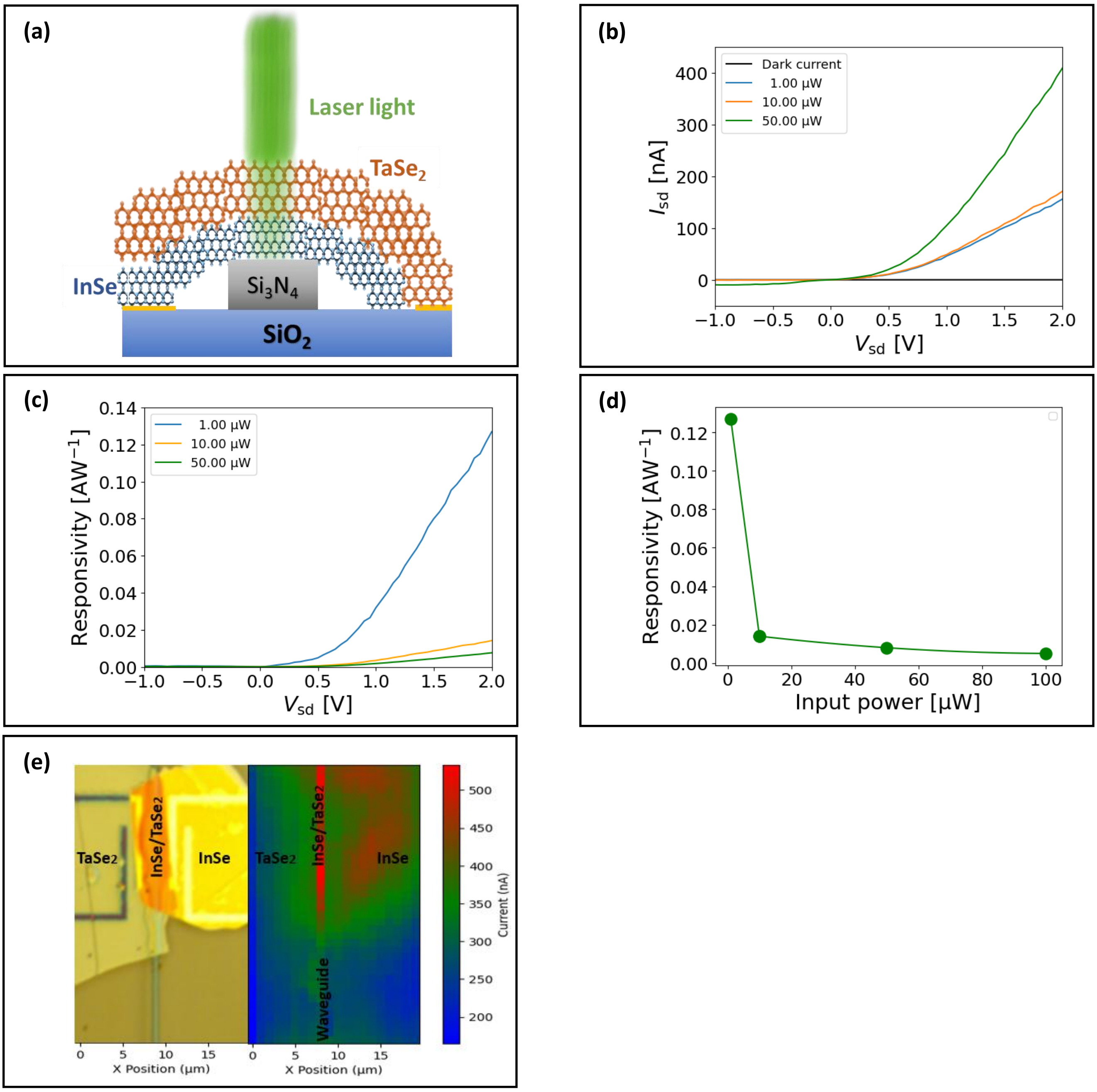

Fig. S7: Illustration and electrical response of InSe/$TaSe_2$ heterostructure with normally incident light onto the heterostructure. All measurements are conducted at a gate voltage of 0 V. (a) 2D schematic diagram of normally incident light from the top onto the InSe/$TaSe_2$ heterostructure on a silicon nitride waveguide and pre-patterned Au/Cr metal contacts. (b) *IV*-characteristics and photocurrent generated for different optical powers. (c) Responsivity as a function of source-drain voltage in the device. (d) Responsivity in the device for different optical powers of a green laser light incident normally from the top at a source-drain voltage of 2 V. (e) Photocurrent mapping in the InSe/$TaSe_2$ heterostructure as the light is coupled normally from the top. The red color indicates the region in the device where the higher amount of photocurrent is generated.

S8: NEP and NPDR response InSe-based and InSe/TaSe$_2$ heterojunction devices with normally incident light onto the photodetector.

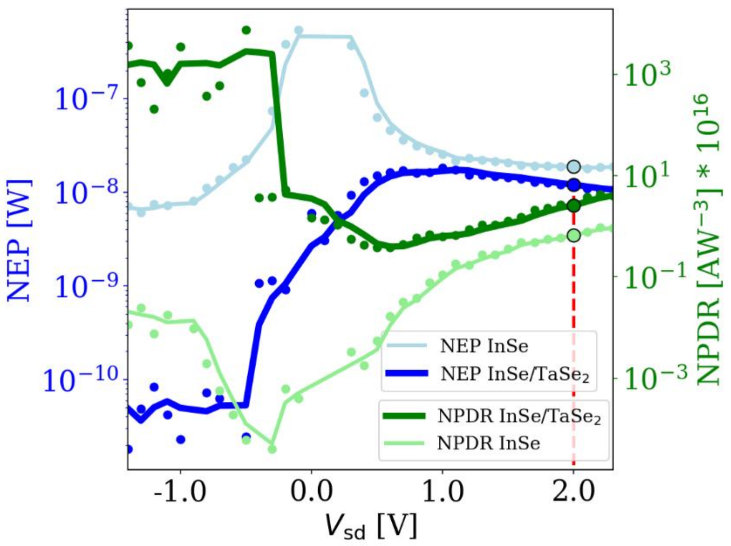

Fig. S8: NEP and NPDR response of InSe-based and InSe/TaSe$_2$ heterojunction devices. The Noise Equivalent Power (NEP) and Noise Power Detection Ratio (NPDR) responses of the InSe-based and InSe/TaSe$_2$ heterojunction devices were analyzed under a source-drain voltage sweep from −1 V to +2 V, with the gate voltage held constant at 0 V.

NEP, which represents the minimum optical power detectable by the device, is a critical performance metric, where lower values indicate improved sensitivity. The graph demonstrates that the NEP values of the InSe/TaSe$_2$ heterojunction device consistently remained lower than those of the InSe-based device across all applied voltage values.

NPDR, on the other hand, measures the detector's ability to differentiate a signal from noise, with higher values indicating superior performance. As shown in the graph, the NPDR values for the InSe/TaSe$_2$ heterojunction heterojunction device were consistently higher than those of the InSe-based device throughout the voltage range.

Together, the NEP and NPDR results highlight the superior performance of the InSe/TaSe$_2$ heterojunction device compared to the InSe-based device. The red vertical line marks the response at a source-drain voltage (V_{sd}) of 2 V, which corresponds to the point where responsivity values were measured.

Fig. S9(a): Benchmark performance comparison showing that our InSe and InSe/$TaSe_2$ photodetectors exhibit superior responsivity than previously reported waveguide-integrated photodetectors based on 2D materials.

Device materials	Material thickness	Device architecture	Incident Wavelength [nm]	V_g / V_{sd}	Responsivity [A/W]	Reference
InSe/$TaSe_2$	20 nm / 20 nm	Waveguide-integrated	532	0 V / 2 V	2.54	This work
InSe	20 nm	Waveguide-integrated	532	0 V / 2 V	1.69	This work
InSe	90 nm	Waveguide-integrated	976	0 V / 5 V	0.38	[1]
MoS_2-InSe	242 nm / 107 nm	Waveguide-integrated	532	-60V/ 2V	0.11	[2]
GQDs/InSe	Not specified	Non-waveguide-integrated	635	Not specified	27.48	[3]
InSe	20 nm<br>100 nm	Non-waveguide-integrated	658	Not specified	7.84<br>0.59	[4]
Surface-Doped InSe	16 nm	Non-waveguide-integrated	470	0 V / 1 V	19 300	[5]
$MoSe_2$-WS_2	20 nm / 1 nm	Waveguide-integrated	780	0 V / 2 V	0.97	[6]
BP-$MoTe_2$	13 nm/ 10.6 nm	Waveguide-integrated	1503	0 V / 0 V	0.27	[7]
$MoTe_2$	35 nm	Waveguide-integrated	1300	0 V / -3 V	0.20	[8]
$MoTe_2$	60 nm	Waveguide-integrated	1550	0 V / -2 V	0.50	[9]
$MoTe_2$ - Graphene	30 nm	Non-waveguide-integrated	1064	0 V / 1 V	970	[10]
Graphene- MoS_2	1 nm	Waveguide-integrated	532	30 V / 0 V	0.44	[11]
MoS_2	0.65 nm	Non-waveguide-integrated	561	-70V/ 8V	0.88	[12]
MoS_2	0.65 nm	Waveguide-integrated	647	0 V / 1 V	1000	[13]
$TaSe_2$-MoS_2-graphene	0.7 nm	Non-waveguide-integrated	532	0 V / 0.2 V	10	[14]
$PdSe_2$-MoS_2	14 nm	Non-waveguide-integrated	10600	0 V / 1 V	42	[15]
Graphene	0.37 nm	Waveguide-integrated	1550	3.2 V / -0.3 V	0.40	[16]
Black phosphorus	40 nm	Waveguide-integrated	2000	0 V / 0.4 V	0.31	[17]
Black phosphorus	40 nm	Waveguide-integrated	3680<br>4000	0 V / 1 V	23<br>2	[18]

Fig. S9(b): Benchmark comparison illustrating the superior responsivity of our InSe and InSe/$TaSe_2$ waveguide-integrated photodetectors relative to previously reported 2D material-based devices.

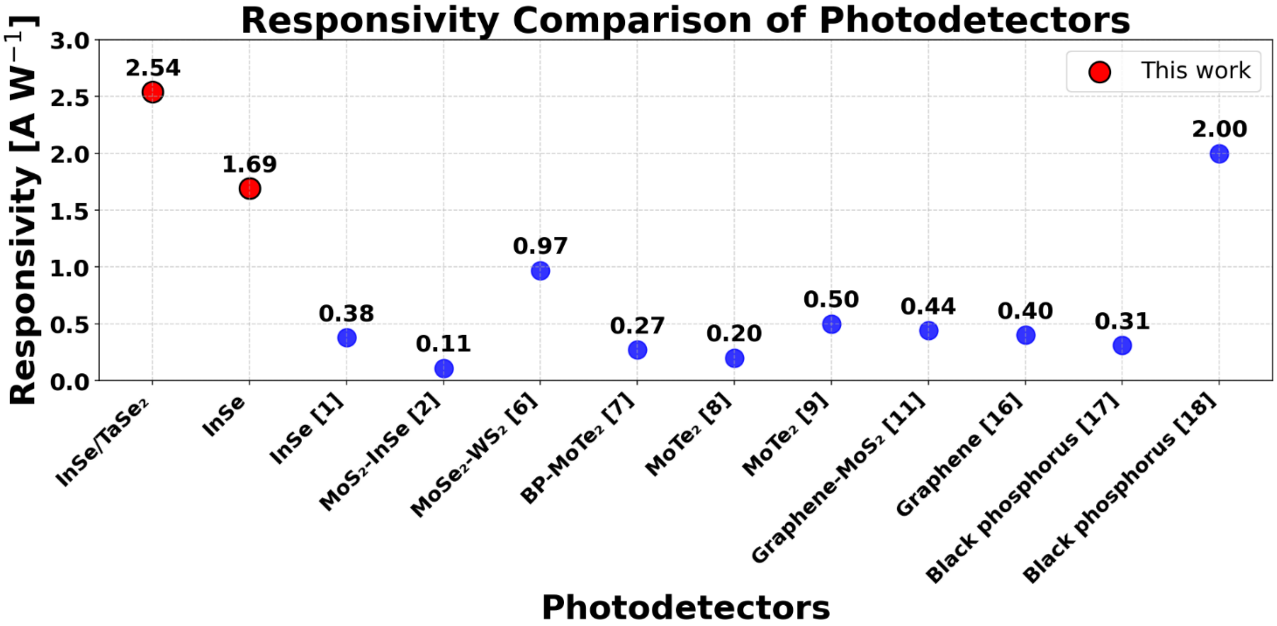

References

1 S. R. Tamalampudi, J. E. Villegas, G. Dushaq, R. Sankar, B. Paredes and M. Rasras, *Adv Photonics Res*, ,DOI:10.1002/adpr.202300162.

2 X. Cui, M. Du, S. Das, H. H. Yoon, V. Y. Pelgrin, D. Li and Z. Sun, *Nanoscale*, , DOI:10.1039/d2nr01042a.

3 W. Wu, Z. Li, X. Liu, et al., *Nanoscale*, DOI:10.1039/D2TC03395J.

4 M. Wasala, P. Patil, S. Ghosh, L. Weber, S. Lei and S. Talapatra, *Oxford Open Materials Science*, **2021**, *1*, itab010, DOI: 10.1093/oxfmat/itab010.

5 H. Jang, Y. Seok, Y. T. Choi, S. H. Cho, K. Watanabe, T. Taniguchi and K. Lee, *Adv Funct Mater*, DOI:10.1002/adfm.202006788.

6 R. Gherabli, S. R. K. C. Indukuri, R. Zektzer, C. Frydendahl and U. Levy, *Light Sci Appl*, DOI:10.1038/s41377-023-01088-4.

7 R. Tian, X. Gan, C. Li, X. Chen, S. Hu, L. Gu, D. Van Thourhout, A. Castellanos-Gomez, Z. Sun and J. Zhao, *Light Sci Appl*, DOI:10.1038/s41377-022-00784-x.

8 N. Flöry, P. Ma, Y. Salamin, A. Emboras, T. Taniguchi, K. Watanabe, J. Leuthold and L. Novotny, *Nat Nanotechnol*, 2020, **15**, 118–124.

9 R. Maiti, C. Patil, M. A. S. R. Saadi, T. Xie, J. G. Azadani, B. Uluutku, R. Amin, A. F. Briggs, M. Miscuglio, D. Van Thourhout, S. D. Solares, T. Low, R. Agarwal, S. R. Bank and V. J. Sorger, *Nat Photonics*, 2020, **14**, 578–584.

10 W. Yu, S. Li, Y. Zhang, W. Ma, T. Sun, J. Yuan, K. Fu, and Q. Bao, *Small*, DOI:10.1002/smll.201700268.

11 Z. Wu, T. Zhang, Y. Chen, Y. Zhang and S. Yu, *Physica Status Solidi - Rapid Research Letters*, 2019, 13.

12 O. Lopez-Sanchez, D. Lembke, M. Kayci, A. Radenovic and A. Kis, *Nat Nanotechnol*, 2013, **8**, 497–501.

13 J. F. Gonzalez Marin, D. Unuchek, K. Watanabe, T. Taniguchi and A. Kis, *NPJ 2D Mater Appl*, DOI:10.1038/s41699-019-0096-4.

14 M. Mahajan, S. Kallatt, M. Dandu, N. Sharma, S. Gupta and K. Majumdar, *Commun Phys*, DOI:10.1038/s42005-019-0190-0.

15 M. Long, Y. Wang, P. Wang, X. Zhou, H. Xia, C. Luo, S. Huang, G. Zhang, H. Yan, Z. Fan, X. Wu, X. Chen, W. Lu and W. Hu, *ACS Nano*, 2019, **13**, 2511–2519.

16 J. Guo, J. Li, C. Liu, Y. Yin, W. Wang, Z. Ni, Z. Fu, H. Yu, Y. Xu, Y. Shi, Y. Ma, S. Gao, L. Tong and D. Dai, *Light Sci Appl*, DOI:10.1038/s41377-020-0263-6.

17 Y. Yin, R. Cao, J. Guo, C. Liu, J. Li, X. Feng, H. Wang, W. Du, A. Qadir, H. Zhang, Y. Ma, S. Gao, Y. Xu, Y. Shi, L. Tong and D. Dai, *Laser Photon Rev*, DOI:10.1002/lpor.201900032.

18 L. Huang, B. Dong, X. Guo, Y. Chang, N. Chen, X. Huang, W. Liao, C. Zhu, H. Wang, C. Lee and K. W. Ang, *ACS Nano*, 2019, **13**, 913–921.
